# Clinical disease characteristics of patients with Niemann-Pick Disease Type C: findings from the International Niemann-Pick Disease Registry (INPDR)

**DOI:** 10.1186/s13023-022-02200-4

**Published:** 2022-02-14

**Authors:** Shaun C. Bolton, Vina Soran, Mercedes Pineda Marfa, Jackie Imrie, Paul Gissen, Helena Jahnova, Reena Sharma, Simon Jones, Saikat Santra, Ellen Crushell, Miriam Stampfer, Maria Jose Coll, Charlotte Dawson, Toni Mathieson, James Green, Andrea Dardis, Bruno Bembi, Marc C. Patterson, Marie T. Vanier, Tarekegn Geberhiwot

**Affiliations:** 1 University Hospitals Birmingham NHS Foundation Trust, Birmingham, UK; 2 University of Birmingham, Birmingham, UK; 3 Hospital Sant Joan de Deu, Barcelona, Spain; 4 International Niemann-Pick Disease Registry, Newcastle, UK; 5 NIHR Great Ormond Street Hospital Biomedical Research Centre, University College London, London, UK; 6 Charles University Prague, Prague, Czech Republic; 7 Salford Royal Foundation NHS Trust, Manchester, UK; 8 Manchester University NHS Foundation Trust, Manchester, UK; 9 Birmingham Women’s and Children’s NHS Foundation Trust, Birmingham, UK; 10 Children’s Health Ireland at Temple Street, Dublin, Ireland; 11 Universitatsklinikum Tubingen Institut fur Medizinische Genetik und angewandte Genomik, Tübingen, Germany; 12 Hospital Clinic de Barcelona, Barcelona, Spain; 13 Regional Coordinator Centre for Rare Disease, AMC Hospital of Udine, Udine, Italy; 14 Mayo Clinic Departments of Neurology, Pediatric and Adolescent Medicine and Medical Genetics, Rochester, USA; 15 INSERM, Lyon, France; 16 Hôpitaux de Lyon, Lyon, France

## Abstract

**Background:**

Niemann-Pick Disease Type C (NPC) is an autosomal recessive rare disease characterised by progressive neurovisceral manifestations. The collection of on-going large-scale NPC clinical data may generate better understandings of the natural history of the disease. Here we report NPC patient data from the International Niemann-Pick Disease Registry (INPDR).

**Method:**

The INPDR is a web-based, patient-led independent registry for the collection of prospective and retrospective clinical data from Niemann-Pick Disease patients. Baseline data from NPC patients enrolled into the INPDR from September 2014 to December 2019 was extracted to analyse the demographic, genetic and clinical features of the disease.

**Results:**

A total of 203 NPC patients from six European countries were included in this study. The mean age (SD) at diagnosis was 11.2 years (14.2). Among enrolled patients, 168 had known neurological manifestations: 43 (24.2%) had early-infantile onset, 47 (26.4%) had late-infantile onset, 41 (23.0%) had juvenile onset, and 37 (20.8%) had adult onset. 10 (5.6%) patients had the neonatal rapidly fatal systemic form. Among the 97 patients with identified *NPC1* variants, the most common variant was the c. 3182T > C variant responsible for the p.lle1061Thr protein change, reported in 35.1% (N = 34) of patients. The frequencies of hepatomegaly and neonatal jaundice were greatest in patients with early-infantile and late-infantile neurological onset. Splenomegaly was the most commonly reported observation, including 80% of adult-onset patients. The most commonly reported neurological manifestations were cognitive impairment (78.5%), dysarthria (75.9%), ataxia (75.9%), vertical supranuclear gaze palsy (70.9%) and dysphagia (69.6%). A 6-domain composite disability scale was used to calculate the overall disability score for each neurological form. Across all with neurological onset, the majority of patients showed moderate to severe impairments in all domains, except for ‘swallowing’ and ‘seizure’. The age at diagnosis and death increased with increased age of neurological symptom onset. Miglustat use was recorded in 62.4% of patients and the most common symptomatic therapies used by patients were antiepileptics (32.9%), antidepressants (11.8%) and antacids (9.4%).

**Conclusion:**

The proportion of participants at each age of neurological onset was relatively equal across the cohort. Neurological manifestations, such as ataxia, dysphagia, and dysarthria, were frequently observed across all age categories.

## Introduction

Niemann-Pick Disease Type C (NPC) is a rare, autosomal recessive disorder resulting from variations to either the *NPC1* or *NPC2* gene. Pathogenic variants in either gene lead to the impaired movement of cholesterol and lipids out of the lysosome and late endosome, resulting in the accumulation of lipids within the affected cell [1]. Advances in diagnostics, disease modifying therapy and disease awareness have led to the recognition of NPC as a heterogeneous disease, ranging from a fatal antenatal disorder to an adult-onset chronic neurodegenerative disease.

The clinical spectrum of NPC encompasses a range of non-specific neurological and systemic manifestations at an early stage and varying by age of onset and organ involvement. Disease onset in early infancy including the neonatal period is typically characterised by visceral manifestations such as neonatal cholestatic jaundice and hepatosplenomegaly with, in a small subset of patients, rapid progression into liver and respiratory failure leading to death [2]. At infancy, childhood or older age, neurological manifestations become prevalent, such as hypotonia, gelastic cataplexy, ataxia, dysarthria, dysphagia, vertical supranuclear gaze palsy (VSGP), seizures and cognitive impairment. Psychiatric symptoms are also prevalent in older NPC patients [2]. NPC is a chronic and progressive disease, with the rate of progression varying between individuals, including between siblings [3]. Age at onset of neurological manifestation is recognised as a predictor of disease progression. The classification of NPC by age of neurological onset is as follows: early infantile (< 2 years) (visceral-neurodegenerative form); late-infantile (2–6 years) and juvenile (6–15 years) (neurodegenerative form); adult (> 15 years) (psychiatric-neurodegenerative form) [4].

The rarity, varied age of onset, heterogeneous and non-specific manifestations of the disease pose significant challenges in understanding the natural course of NPC. Observational studies of NPC patients across developed nations have been reported, with data from France, Spain, the UK and USA [5–10]. These studies have advanced the understanding of NPC as a spectrum disorder, leading to enhanced care for NPC patients. The consolidation of existing data alongside the generation of new data via an international patient registry may lead to the improved characterisation of NPC.

Here we report the findings of an analysis of Niemann-Pick Disease Type C data gathered through the International Niemann-Pick Disease Registry. We have developed an exemplar patient led independent international registry capable of providing an inventory of patients for recruitment to observational and interventional studies.

## Results

### Demographics, patient characteristics, biochemical and genetic diagnostics

A total of 203 patients were enrolled from 11 NPD centres across 6 countries: The Czech Republic, Germany, Ireland, Italy, Spain and the UK. A summary of patient demographics are given in Table 1. The study participants were equally split in sex, with 49.8% male and 50.2% female. 39.9% of patients were prospectively recruited. The mean (SD) age at diagnosis was 11.2 (14.2) years. Of the 203 patients enrolled, 168 patients had known neurological manifestations at the point of data collection. 43 (24.2%) had early-infantile onset, 47 (26.4%) had late infantile onset, 41 (23.0%) had juvenile onset, and 37 (20.8%) had adult onset (Table 2). Of the remaining 35 patients, 10 (5.6%) had the neonatal rapidly fatal form, 21 patients were reported as not having neurological manifestations, and 4 patients having visceral symptoms only.

**Table 1:** Demographics of patients with NPC

Characteristic	N (%)	Mean (SD)
*Overall population (n = 203)*		
Male	101 (49.8)	
Female	102 (50.2)	
Age at diagnosis	203	11.2 (14.2)
Age at enrollment	203	25.8 (16.5)
Prospective patient records	81 (39.9)	
Retrospective patient records	122 (60.1)

**Table 2:** Classification of patients (n = 182) into NPC clinical forms

Clinical form	N (%)	Mean age at diagnosis (SD) and range in years
Neonatal systemic rapidly fatal (< 28 days)	10 (5.6)	0.37 (0.25)—0.0–0.9
Early infantile neurological (< 2 years)	43 (24.2)	2.16 (2.75)—0.0–17
Late infantile neurological (2 to < 6 years)	47 (26.4)	4.92 (3.25)—0.1–12
Juvenile neurological (6 to < 15 years)	41 (23.0)	9.89 (5.34)—0.1–22
Adult neurological (> 15 years)	37 (20.8)	34.12 (13.84)—1.4–68
No neurological manifestations	4	19 (17.95)—0.7–42

In this cohort comprising of 60% retrospective cases, a number of patients had been diagnosed by filipin test and early rate of LDL-induced cholesteryl esterification [2, 11], and DNA testing was recorded for only 98 patients; among them 97 presented biallelic variants in *NPC1* gene and 1 patient had variants in *NPC2* gene (Table 3). Regarding *NPC1* gene variants, an important heterogeneity in the genotypes was observed and the majority of patients were compound heterozygous. The most common *NPC1* variant was the c.3182T > C (p.lle1061Thr) variant related with the classical biochemical phenotype and with the juvenile clinical form of presentation. The p.I1061T variant was reported in 28.6% (N = 28) of patients, with a total number of 28 alleles. Secondly, the c.3019C > G (p.P1007A) variant was reported in 10.2% of patients (N = 10) and related with the variant biochemical (filipin) profile and with the juvenile-adult forms of the disease. A total of 9 alleles were reported for the variant. Finally, the c.3557G > A (p.R1186H) variant was reported in 9.2% of patients (N = 9). Plasma levels of Cholestane-triol were documented in 27 patients, 23 of them presented levels above the cut-off, 2 showed borderline concentrations and 2 displayed normal values.

**Table 3:** Diagnostic data of NPC patients

	N (%)
**NPC1 sequencing (n = 97)**	
c.3182T > C (p.I1061T)	28 (28.6)
c.3019C > G (p.P1007A)	10 (10.2)
c.3557G > A (p.R1186H)	9 (9.2)
**Cholestane triol levels (n = 27)**	
Elevated	23 (85)
Borderline	2 (7)
Normal	2 (7)
**Neuroimaging MRI (n = 49)**	
Normal	29 (59)
Abnormal	20 (41)
Cortical atrophy	10 (50)
Cerebellar atrophy	9 (45)
White matter change	5(25)
**CT (n = 7)**	
Normal	6 (86)
Abnormal—cortical atrophy	1 (14)

### Clinical features

#### Systemic manifestations

**Fig. 1:**
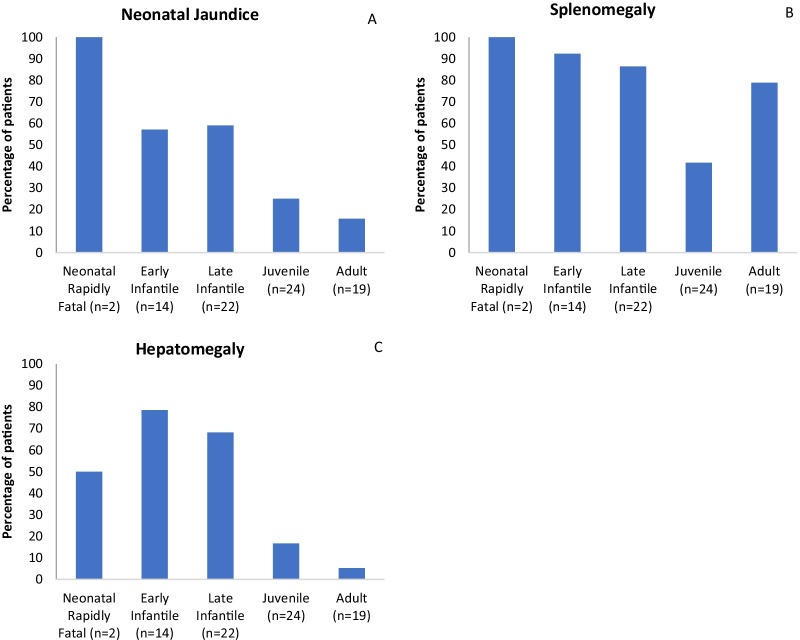
**A**–**C** Percentage of patients with **A** neonatal jaundice, **B** splenomegaly, and **C** hepatomegaly. N = total number of patients in category

Systemic manifestations consisting of neonatal jaundice, spleen and liver enlargement and liver failure was recorded for 81 patients and are summarised in Fig. 1. The proportion of patients who developed splenomegaly was 72.8%, whilst hepatomegaly was reported in 39.5% of patients. Similarly, neonatal jaundice was noted in 39.5% of patients, and hepatic failure in the neonatal period was 6.2%. The proportion of patients with neonatal jaundice, spleno- and hepatomegaly decreased with increasing age of neurological onset.

#### Neurological manifestations

**Fig. 2:**
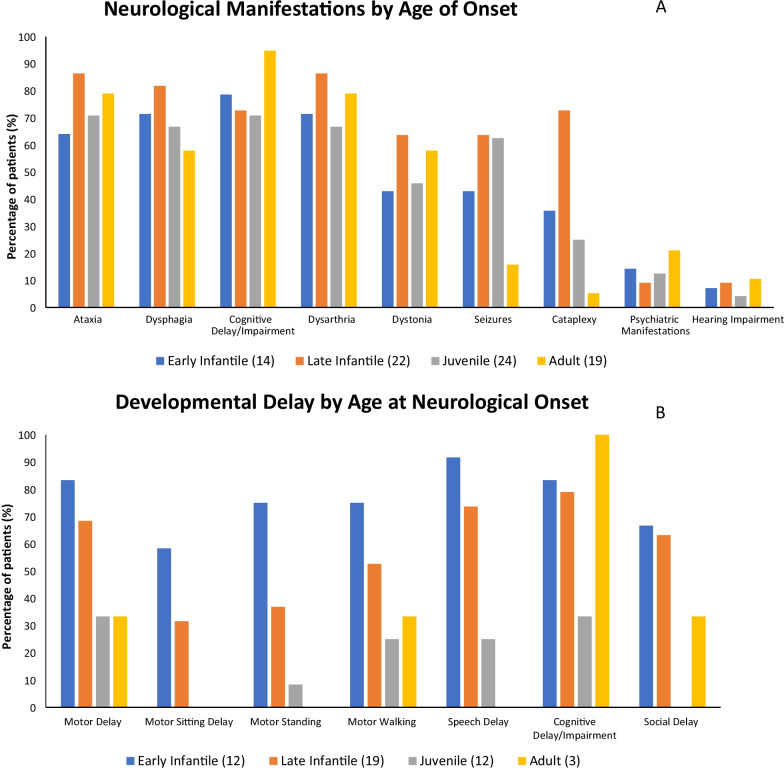
Neurological manifestations in relation to age at neurological onset (**A**). Developmental delay in relation to age of neurological onset (**B**). N = total number of patients in category

The profiles of patients with neurological manifestations according to the age at neurological-onset is summarised in Fig. 2A. The most common neurological manifestations reported include cognitive impairment (78.5%), dysarthria (75.9%), ataxia (75.9%), vertical supranuclear gaze palsy (VSGP) (70.9%), dysphagia (69.6%), dystonia (53.2%), seizures (48.1%), and cataplexy (35.4%). Overall, the late infantile onset subgroup made up the highest proportion of neurological manifestations, with the exception of psychiatric manifestations and cognitive impairment. The latter two components were predominant in adult onset phenotype. The least prevalent neurological manifestations were psychiatric manifestations (13.9%) and hearing impairment (7.6%).

Developmental milestone data stratified according to age at neurological onset is summarised in Fig. 2B. Of the 46 patients where data is available, the most common manifestations were delay/impairments in: cognition (69.6%), speech (60.9%), motor (60.9%) and social (45.7%). In respect to neurological classification, speech delay was most common in early infantile (91.7%), whilst cognitive impairment was most common in late infantile (78.9%) and adult (100%), with motor delay (33.3%) and cognitive impairment (33.3%) predominant in juvenile onset.

Neuroimaging assessment data was available for 49 patients (Table 1). A normal MRI result was reported in 29 patients (59.2%). Abnormal MRI results were reported for 20 patients (40.8%), with 7 patients reporting 2 or more MRI findings. The most common abnormal findings were: cortical atrophy (N = 10), cerebellar atrophy (N = 9), and white matter changes (N = 3). CT scan results were available for 7 patients, where normal results where shown for 6 patients, and 1 patient shown to have an abnormal result with cortical atrophy.

### Disease stage and progression

**Fig. 3:**
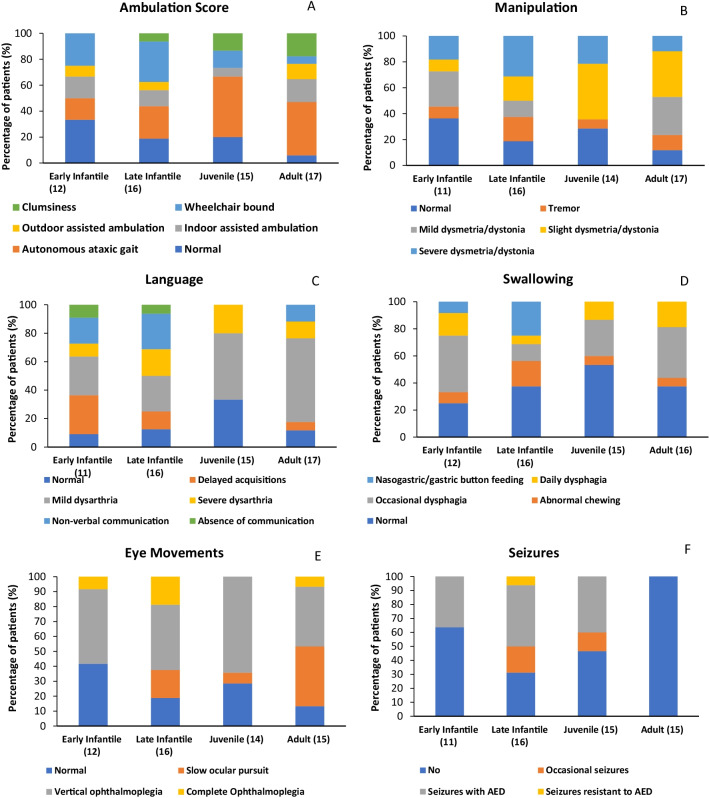
**A**–**F** Disability scale scores stratified by age at neurological onset. N = total number of patients in category

An assessment of disease severity was performed using a 6-domain disability scale [7] to calculate the overall disability score according to the age at neurological-onset (Fig. 3). A total disability score was calculated for each patient by combining their ambulation, manipulation, speech, swallowing, eye movement, and seizure scores. Each subcategory was scored from normal to most severe based on the pre-defined validated disease progression domains.

**Fig. 4:**
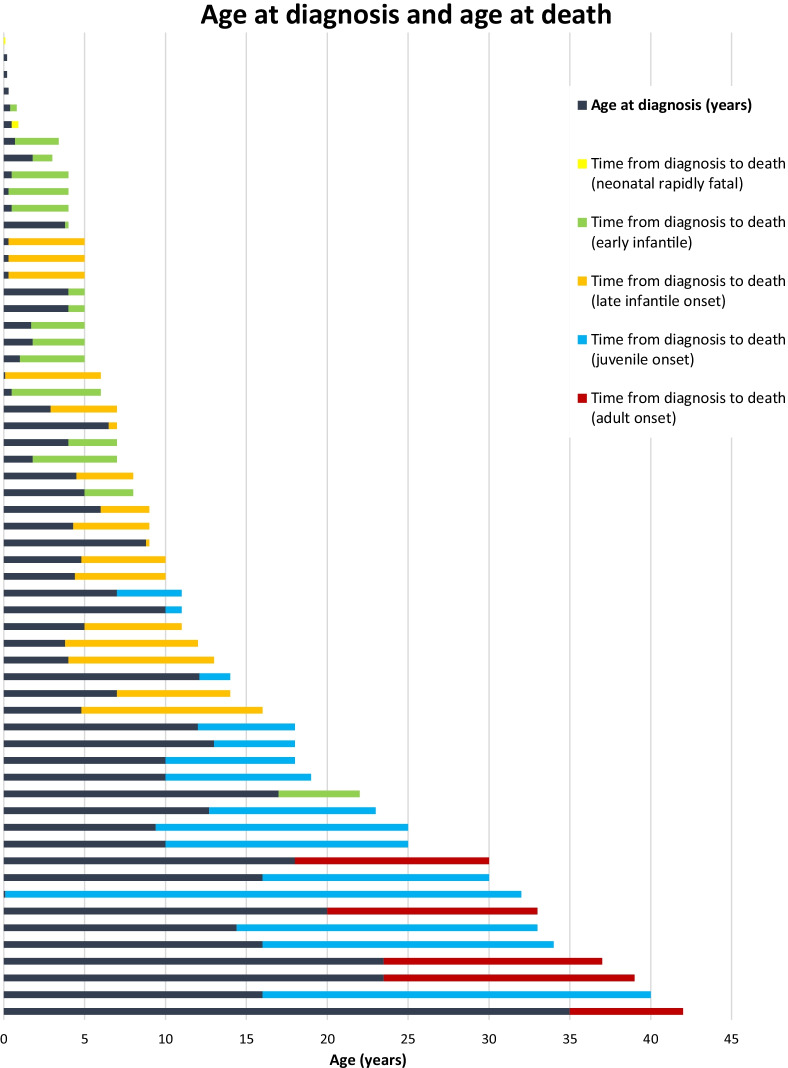
Age at diagnosis and death according to disease onset. Total number of patients = 59

Data on the age at diagnosis and age at death was available for 59 patients. Figure 4 provides an overview of patient data illustrating the recorded age at diagnosis, and the subsequent age at death, stratified according to the age of neurological onset classification. In patients with the early infantile form (N = 17), the median ages at diagnosis and death were 1.8 years (0.3–17.0) and 5 years (0.8–22.0), respectively. In patients with the late infantile form (N = 17), the median ages at diagnosis and death were 4.4 years (0.1–8.8) and 8.8 years (5.0–16.0) respectively. In the juvenile onset group (N = 15), the median ages at diagnosis and death were 12.0 years (0.1–16.0) and 23.0 years (11.0–40.0) respectively. In patients with adult onset (N = 5), the median ages at diagnosis and death were 23.5 years (18.0–35.0) and 37.0 years (30.0–42.0) respectively. As anticipated, the age at diagnosis and death increased with increased age at neurological onset.

### Treatment modalities

Data describing the symptomatic therapies used prior to or at enrolment was available for 85 patients. The mean total number of therapies used by each patient was 1.8. The most common symptomatic therapies used by patients were antiepileptics (32.9%), antidepressants (11.8%) and antacids (9.4%). The disease modifying therapy, Miglustat, was used by 62.4% of patients and the mean duration of use was 48.7 months. Diarrhoea was the most frequently reported adverse effect occurring in 56% of cases. Low platelet count was reported in 19% of cases and tremor was reported in 13% of cases.

## Discussion

As NPC is a rare disease, patient population sizes are limited and tracking the natural history of the disease and enrolling patients for a clinical study is very challenging. The INPDR is a patient led registry designed to: (a) better understand the natural history of Niemann-Pick disease worldwide; (b) provide an inventory of patients for recruitment to observational and interventional studies; (c) establish genotype–phenotype correlations; (d) provide support for education of health professionals and empowerment of patients. Data from 203 NPC patients from 6 countries were available for analysis, forming one of the largest cohorts of patients with NPC reported to date.

The p.I1061T variant was the most common variant in this population, with detection in 28.6% of patients. This aligns with previous research, where p.I1061T is most common in Western European decent populations [12] with varied frequency as reported in cohort studies from the United Kingdom [8], Spain [13], the Czech Republic [11], and Italy [14]

Disease classification based on the age of onset of the first neurological symptoms has been accepted as a guide to clinicians in providing day to day care, genetic counselling and to estimate the trajectory of the disease course [12]. The proportion of participants at each age category based on neurological onset was relatively consistent; 24.2% early infantile, 26.4% late infantile, 23.0% juvenile and 20.8% adult patients. These findings are in line with the previously reported observational studies [8, 10]. The type and onset of visceral symptoms varied across the age groups with decreased occurrence with an increasing age. Neonatal jaundice and hepatomegaly were most prevalent in early infantile and late infantile patients. As has been reported in prior NPC cohort studies, splenomegaly is the most consistent observation across the age spectrum and was recorded in 80% of adult patients [7, 8, 10, 11].

Neurological signs such as ataxia, dysphagia, dysarthria and cognitive impairment were the most common, being reported in over two thirds of patients from all age categories. Similar to visceral manifestations, some of the neurological findings vary with age at disease onset. It is notable that the frequency of seizure and cataplexy is most common in infantile and juvenile onset and less so in adult. Developmental milestones delays were the most commonly reported neurological observation in early and late infantile forms. In contrast, psychiatric manifestations and hearing impairments are more common in adult onset. These findings are in agreement with previous observations [15]. Neuroimaging data gathered primarily from juvenile and adult individuals showed a variable pattern, with some being normal, while most patients imaging results demonstrate cortical and cerebellar atrophy as the most common changes. These changes have been reported to correlate with measures of ataxia and ocular-motor function [16].

Clinical assessment of disease burden and rate of progression in time or regression with disease modifying therapy is challenging when dealing with progressive diseases such as NPC. We used a modified 6-domain functional disability score [7] as calculated by combining ambulation, manipulation, speech, swallowing, eye movement and seizure scores. The composite disability scores were notably higher in patients with early onset neurological signs, particularly in late infantile patients. This is consistent with the natural history of the disease, which has more severe presentation and rapid disease progression in patients whose neurological manifestations appear at a younger age [2]. On the other hand, juvenile and adult-onset patients had fewer ‘normal’ individual disability scores for ambulation, manipulation and language domains. Eye movements and seizure seem to show the least variability indicating as less sensitive indicators to assess disease progression.

Mortality data was available for 59 patients and the duration of survival after disease onset varies with age spectrum of the disease. As expected, death on average resulted in less than 1 year after onset with the neonatal rapidly fatal forms. Interestingly, we observed a unique pattern with the other three age categories of the disease as follows: early infantile, late infantile and juvenile form, where the mean rate of survival in years doubles for each category of NPC forms. In addition, similar longer survival trend was noted in adult forms. The lack of doubling effect in adults may well be due to delayed diagnosis which in turn hampers the recognition and/or association of early subtle NPC related neuropsychiatric signs. The pattern of disease onset and survival observed in this study is largely in agreement with the findings from the French cohort [5]. This observation can be used to council patients and families about the disease course and prognosis at the time of diagnosis.

Use of various therapeutic modalities was reported in this registry. The mean total number of therapies used by each patient was 1.8. The most common therapy was Miglustat, a potential disease modifying therapy specifically indicated for NPC, reported in 85 (62.4%) patients, with a mean duration of just over 4 years. Other symptomatic therapies were antiepileptic (32.9%), antidepressants (11.8%) and antacids (9.4%). These levels of therapeutic intervention in NPC are in line with the current NPC international clinical management guidelines [4].

This study has several limitations: (1) being an observational, non-interventional real-world study the dataset had some gaps in data points which may reflect variation in standard of care practice and needs of the patient. Therefore, some parameters were analysed with the denominator as “n” rather than 203 patients. (2) The data is a snapshot of the registry, with baseline data reported. Therefore longitudinal follow-up data should be considered to investigate the possible changes in disability score or neurological manifestations in line with existing and future disease modifying treatment. (3) Data reported is from 6 European countries, and may reflect the disease characteristics of patients from those countries rather than being representative of patients globally. The capturing of clinical data from patients beyond these 6 countries may indicate differences to the findings of this analysis. (4) Results from genetic testing were available for only 98 patients, and are therefore not representative of the current patient's population of the registry. Of note, this does not affects the reliability of the diagnosis in other patients, since for a long time, the gold standard diagnostic test for NPC has been functional, based on demonstration of impaired intracellular processing of unesterified cholesterol. As well demonstrated in cohorts from Czech Republic [11], Spain [13] and Italy [14], later genotyping of such patients has well demonstrated the reliability of the historical procedure. The strength of this study lies on the use of the second largest number of NPC patients studied to date and its international scope. Above all, we have developed an exemplar patient led, independent international registry capable of delivering on the unmet need of not only Niemann-Pick Disease but also serves as a model for other rare and neglected disorders.

In summary, the data of 203 NPC patient records captured via the INPDR highlights key patient and disease characteristics that can be tracked for future international studies. To our knowledge, this is the first patient led registry of such international reach. Long term longitudinal data utilising the INPDR on a larger international cohort is currently underway.

## Methods

### Study design and population

The International Niemann-Pick Disease Registry (INPDR) is a multi-centre, multi-national observational patient registry for the collection of Acid Sphingomyelinase Deficiency (ASMD) and NPC patient clinical data. The INPDR was launched in 2013 with the support of a European Commission grant and was developed through the involvement of expert clinicians, researchers and patient advocates from the global Niemann-Pick Disease community. The aim of the INPDR was the creation and maintenance of a web-based electronic data capture (EDC) platform to collect clinical and patient reported data specific to Niemann-Pick Diseases (NPD). The EDC platform was developed by Sleek Ltd (Melbourne, Australia) and launched in 2014.

All patients with a confirmed diagnosis of NPD are eligible to be enrolled into the INPDR. Clinical data was captured using standard of care clinic visit information as source data, with assessments determined by the patient’s clinician. Patients were recruited from six European countries, with research ethics committee approval obtained for all participating centres in line with applicable national and institutional research governance standards of the participating centres and the International Conference on Harmonisation Good Clinical Practice (ICH-GCP) guidelines.

### Data points and data collection

Data points were produced by a committee of expert clinicians, researchers and patient advocates and were adopted during a Scientific Advisory Committee meeting in 2013. The data points were grouped into the following data forms: patient demographics, biochemical (including filipin and biomarkers) and genetic testing, family history, clinical history, physical findings, treatment, disease severity scale, and additional investigations.

For analysis, patients were stratified following the classification into 4 categories based on age at neurological onset, as defined and used in many preceding studies [2, 4, 8–11, 14], plus an additional 5th category for patients with the neonatal systemic rapidly fatal form as delineated in cohorts from the UK, the Czech Republic and Italy [8, 11, 14]. This data point was collected by requiring study clinicians to classify participants in accordance with the categories.

All data was captured via a secure web-based INPDR EDC platform that allows clinicians and their authorised staff to enter clinical data of consented patients. The EDC platform IT infrastructure is currently maintained by OpenApp Ltd (Dublin, Ireland).

In line with the registry protocol, all collected data reflects the patient’s standard of care practice as per the national clinical guidance. No assessments beyond standard of care were mandated by the protocol. Clinical data was entered by the centre’s clinician or a designated member of the clinician’s staff. The baseline data was considered as data entered on or close to the informed consent date.

### Statistical analysis

An extract of NPC baseline data from September 2014 to December 2019 was obtained from the EDC system. Data was extracted into a Microsoft Excel© file format (Microsoft Excel 2010, Microsoft Corporation). The descriptive data analysis was conducted using Microsoft Excel© to obtain the median and range of data points using the median formula (= MEDIAN(range)), the minimum value formula (= MIN(range)), and maximum value formula (= MAX(range)). A CountIf formula (= COUNTIF(range, criteria)) was used to calculate the number of responses associated with a data point. For example, to count the number of ‘Yes’ responses to a data point, the formula = COUNTIF(range, “Yes”) was used. Continuous variables are summarized using descriptive statistics including mean, standard deviation (SD), median, range and 95% confidence interval (CI) of the mean. Categorical variables are summarized using counts and percentages. All summary statistics and percentages were calculated relative to number of patients with available data. Denominators for analysis were the numbers of patients with the corresponding data available and hence different parameters may have different denominators.

## Data Availability

The datasets analysed during the current study are available from the corresponding author on reasonable request.

## Abbreviations

EDC: Electronic data capture
INPDR: International Niemann-Pick Disease Registry
NPC: Niemann-Pick Disease Type C
NPD: Niemann-Pick Disease
VSGP: Vertical supranuclear gaze palsy
